# Soybean Variety Saedanbaek Confers a New Resistance Allele to *Phytophthora sojae*

**DOI:** 10.3390/plants12233957

**Published:** 2023-11-24

**Authors:** Hee Jin You, Kyu-Chan Shim, In-Jeong Kang, Ji-Min Kim, Sungtaeg Kang, Sungwoo Lee

**Affiliations:** 1 Department of Crop Science, College of Agriculture and Life Sciences, Chungnam National University, Daejeon 34134, Republic of Korea; heejinyou0410@gmail.com (H.J.Y.);; 2 Division of Crop Cultivation and Environment Research, Department of Central Area Crop Science, National Institute of Crop Science, Suwon 16613, Republic of Korea; fairjung@korea.kr; 3 Department of Crop Science and Biotechnology, College of Bioresource Science, Dankook University, Cheonan 31116, Republic of Korea; jmkim1206@naver.com (J.-M.K.); kangst@dankook.ac.kr (S.K.)

**Keywords:** soybean, disease resistance, *Phytophthora sojae*, genetic mapping, *Rps*

## Abstract

Phytophthora root and stem rot (PRSR) disease results in substantial losses in soybean production worldwide. The occurrence of PRSR caused by *Phytophthora sojae* Kaufmann & Gerdemann has become increasingly important for soybean production in the Republic of Korea, but domestic soybean–*P. sojae* interaction has been less studied. The disease has been managed by developing varieties harboring resistance to the *Phytophthora sojae* (*Rps*) gene. The present study aimed to identify a major gene locus conferring resistance to new *P. sojae* isolate 2858 in the recombinant inbred line population derived from a cross between parental lines ‘Daepung’ (susceptible) and ‘Saedanbaek’ (resistant). Seventy-three recombination inbred lines (RILs) were evaluated for resistance to *P. sojae* isolate 2858. A resistance locus was identified in the approximate 3.3–4.3 megabase pair region on chromosome 3 using both single-marker and linkage analyses. The *Rps* of Saedanbaek (*RpsSDB*) was located on the well-known *Rps* gene/allele cluster region, which also partially overlapped with a locus previously identified in the Korean soybean variety, ‘Daewon’, resistant to another *P. sojae* isolate 2457 (*RpsDW*). Approximately 402 kilobase pairs of the interval region overlapped, including six nucleotide-binding site-leucine-rich repeat (NBS-LRR)-coding genes. Additional phenotypic assays revealed that Saedanbaek was susceptible to isolate 2457 and that Daewon was susceptible to isolate 2858, indicating that *RpsSDB* and *RpsDW* are different genes or alleles that confer race-specific resistance to the two *P. sojae* isolates. These results provide information that will be helpful for breeders developing *P. sojae*-resistant cultivars.

## 1. Introduction

Soybean [*Glycine max* (L.) Merr.] is one of the most economically valuable crops and an important source of protein and oil worldwide. Soybean production is affected by biotic and abiotic stressors. Phytophthora root and stem rot (PRSR), caused by the soil-borne pathogen, *Phytophthora sojae* Kaufmann & Gerdemann, is one of the most prevalent diseases leading to yield loss in soybean production [1]. Approximately 11 million tons of annual yield loss of soybean due to diseases was estimated in the United States and Canada from 2010 to 2019 [1,2]. Soybean is the primary host of the causal agent *P. sojae*, known to have a narrow host range [3,4]. It is a self-fertile oomycete that usually overwinters as oospores. Sporangia develop when soils are saturated, in which many zoospores will form. Once zoospores are released in soil water, they are attracted to soybean roots by chemotaxis [5]. It eventually encysts, germinates, and invades the root [4]. The symptoms of PRSR in the susceptible soybean include seed decay and seeding damping-off during the early growth stage, and brown stem lesion and collapsing tissue at the later season.

Soybean–*P. sojae* interaction has been most extensively studied in the U.S. in the past several decades [3]. Genetic diversity in the *P. sojae* population is distinguishable among multiple states of the U.S. and the frequency changes of diverse pathotypes were discovered through periodical state-wide studies over decades [3]. Similarly, the diversity and shift of pathotypes of the *P. sojae* population vary by country and by geographical region within a country based on the phenotypic assays using differential varieties and molecular analyses in China, Japan, and Brazil [6,7,8]. The development of resistant cultivars is the most effective way of preventing PRSR [7]. PRSR is mainly controlled by a single qualitative gene, the so-called resistance to the *P. sojae* (*Rps*) gene, and quantitative resistance has been reported as an alternative regulatory mechanism [3]. *Rps*-mediated resistance shows race-specific features, which are determined by the gene-for-gene relationship between the *Rps* of soybean and *avirulence* (*Avr*) gene of *P. sojae* [9,10]. Thus, identification of *Rps* loci and race-specific resistance alleles is essential for developing resistant varieties of *P*. *sojae*. Particular *Rps* genes were preferred, selected, and employed in newly developed resistant soybean varieties based on the genetic diversity of the local *P. sojae* population [7,11].

To date, more than 30 *Rps* alleles have been identified on the 10 chromosomes of soybean using bi-parental genetic mapping [12,13]. These *Rps* loci are not evenly distributed in the genome but tend to cluster in a few genomic regions [12,14]. Among these loci, approximately 20 *Rps* alleles have been mapped to the *R*-gene-rich region on chromosome 3 (*Rps1a*, *1b*, *1c*, *1d*, *1k*, *7*, *9*, *UN1*, *Yu25*, *YD25*, *YD29*, *HN*, *Q*, *WY*, *HC18*, *X*, *GZ*, unnamed in cv. *Waseshiroge* and *Daewon, T1/T2/T3*, and *14*) [15,16,17,18,19,20,21,22,23,24,25,26,27,28,29,30,31]. These alleles were mapped to a range of regions harboring many nucleotide-binding site-leucine-rich repeats (NBS-LRRs or LRR) and serine/threonine protein kinase (STK) genes, which are primarily known as disease resistance genes in plant species [32,33]. Other loci were mapped on chromosome 2 (*RpsZS18*), 7 (*Rps11*), 10 (*RpsSu*), 13 (*Rps3a*, *3b*, *3c*, *8*, *SN10*, and *CD*), 16 (*Rps2* and *UN2*), 17 (*Rps10*), 18 (*Rps4*, *5*, *6*, *JS*, *12*, and *13*), and 19 (*RpsYB30*) [16,18,34,35,36,37,38,39,40,41,42,43,44,45,46].

In the Republic of Korea, PRSR was initially reported in the Chungchengnam-do in the late 1990s [47]; however, only a few studies were conducted in recent years. Twenty soybean cultivars widely planted in the Republic of Korea were screened to identify *Rps*-resistant cultivars against four isolates (P-9662, P-98145, 2457, and 3444-1) [48]. Among the cultivars, ten showed susceptible reactions to each isolate, and isolate-specific resistance reactions were observed in 11 varieties. ‘Daewon’ shows *Rps*-mediated resistance to isolate 2457 and the resistance gene was detected on chromosome 3 using linkage analysis of a recombination inbred line (RIL) population [24]. Another resistant variety, Socheong2, displayed resistance to *P. sojae* isolates 40412 and 2457 [49]. Linkage mapping revealed three *Rps* loci on chromosomes 3 and 18, and two at distant genomic locations on chromosome 18 [49].

Soil-borne pathogens such as *P. sojae* and *Fusarium* spp. occur more frequently under wet soil conditions [3,4]. PRSR is more frequently observed during the growing season because of increased soybean cultivation in converted paddy fields, where soil moisture levels tend to be high, owing to poor drainage. Consequently, PRSR has become a serious problem for soybean production in the Republic of Korea [48]. A preliminary screening showed that the cultivar ‘Saedanbaek’ is resistant to *P. sojae* isolate 2858, which has not yet been genetically characterized for the resistance. Thus, this study aimed to identify a genomic region associated with *R*-gene-mediated resistance to *P. sojae* isolate 2858 using an RIL population derived from a cross between ‘Daepung’ and Saedanbaek.

## 2. Results

### 2.1. Phenotypic Assay for Resistance to P. sojae

**Figure 1:**
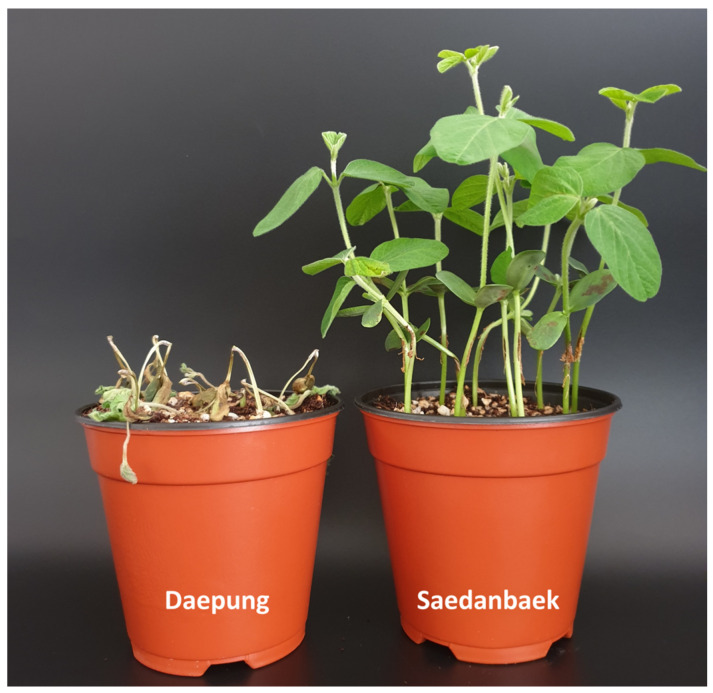
Phenotypic reactions of Daepung and Saedanbaek following inoculation with *Phytophthora sojae* isolate 2858.

The two parents, Daepung and Saedanbaek, showed typical susceptibility and resistance reactions to *P. sojae* isolate 2858 in repeated experiments (Figure 1). Of the RILs, 25 and 43 exhibited resistance and susceptibility, respectively (Table 1). Intermediate reactions were observed in four RILs, with 45–60% dead seedlings. A goodness-of-fit test demonstrated that the segregation ratio agreed marginally with the expected ratio in the F_6_ generation (34.9:2.2:34.9 for resistance: intermediate: susceptibility), suggesting that the trait was mediated by a single gene (χ^2^ = 6.05, *p* = 0.05) (Table 1). 

### 2.2. Single-Marker Analysis of Variance (ANOVA)

Singe-marker ANOVA was performed to detect significant marker-trait associations for 26,130 SNP. In total, 28 SNP markers were highly significant and located in a genomic region of 3.3–4.3 megabase pairs (Mbp) on chromosome 3, for which *p*-values ranged from 3.7 × 10^−16^ to 4.4 × 10^−23^ (Table 2). The estimated *R*^2^ ranged from 71% to 82%, indicating that the resistance was controlled by a single major gene at this locus (Table 2). No SNP was significant on the other chromosomes based on the threshold of *p* < 3.8 × 10^−7^.

### 2.3. Identification of a Resistance Locus Using Linkage Analysis

**Figure 2:**
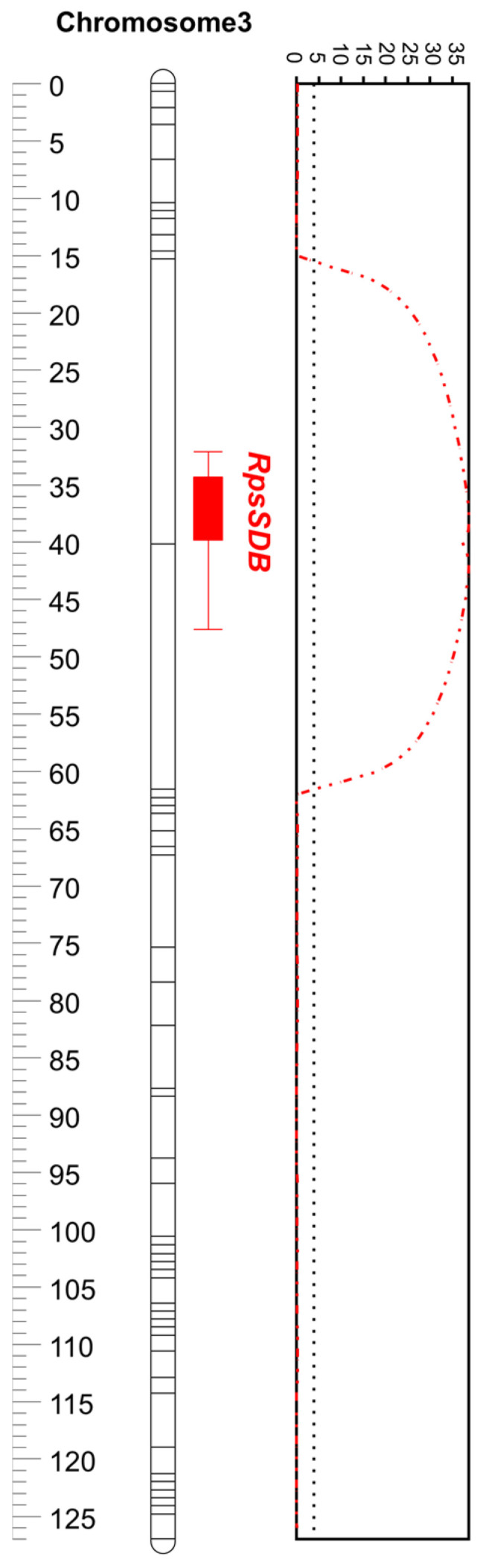
The genomic region for resistance to *Phytophthora sojae* isolate 2858 identified on chromosome 3 in the Daepung × Saedanbaek population. The bands on the chromosome indicate that SNP marker loci integrated into the genetic map. The LOD graph to the right of the chromosome show the most likely position of the resistance gene locus. The hatched line on the LOD graph indicates the LOD threshold. The 1- and 2-LOD intervals are displayed as bar and loid lines, respectively.

In total, 963 SNPs evenly distributed over 20 chromosomes were retained after binning 26,130 SNPs, which were subsequently used to construct genetic maps. The number of SNPs that were integrated into each chromosome ranged from 33 to 63, with an average of 48 (Table 3). The total length of the genetic map was approximately 1586 cM, with an average marker interval of 1.7 cM (Table 3). In the genetic bin map, the genetic positions of the mapped SNPs were in good agreement with the physical positions based on the reference, Glyma.Wm82.a2 (Figure S1). Linkage analysis identified the same genomic region (3.3–4.3 Mbp) with an extremely high logarithm of odds (LOD) of 38.7, explaining 93% of the phenotypic variance (Table 4, Figure 2). The peak of LOD was located at the AX-90404495 on the genetic bin map, which represented all the 28 significant SNPs detected by ANOVA. The additive effect of the locus was 45.5, indicating that the Saedanbaek allele conferred resistance to *P. sojae* (Table 4).

### 2.4. Comparison of the Rps Locus of Saedanbaek (RpsSDB) with That of Daewon (RpsDW)

**Figure 3:**
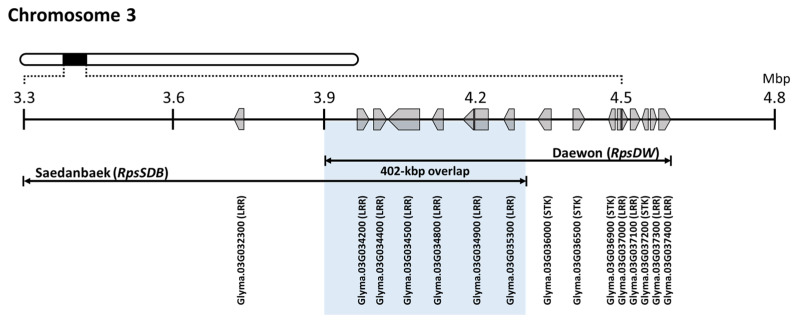
Comparison of the identified *Rps* loci between Saedanbaek (*RpsSDB*) and Daewon (*RpsDW*). Several nucleotide-binding site-leucine-rich repeat (NBS-LRR)- and serine/threonine protein kinase (STK)-coding genes were annotated in this interval.

The resistant allele of Saedanbaek identified in the present study was named *RpsSDB* to distinguish it from other colocalized *Rps* alleles (Figure 3). *RpsSDB* partially overlapped with *RpsDW* detected in ‘Daewon’, which was previously mapped on chromosome 3 [24]. To distinguish between the two *Rps* loci, recombinants within the target interval were screened using RIL populations. As RILs with recombination breaks within the *RpsSDB* were not detected in the Saedanbaek population, *RpsSDB* and *RpsDW* overlapped in an approximately 402 kbp interval (3,893,390–4,295,128 bp) (Figure 3). According to the soybean reference genome (Glyma.Wm82.a2.v1), 53 and 31 genes, in total, were annotated in the interval of *RpsSDB* and *RpsDW* intervals, including seven and eight copies of NBS-LRR- or STK-coding genes, the two main categories of disease resistance genes, respectively (Table S1). The overlapped interval included six LRR-coding genes, i.e., Glyma.03G034200, Glyma.03G034400, Glyma.03G034500, Glyma.03G034800, Glyma.03G034900, and Glyma.03G035300 (Figure 3, Table S1).

### 2.5. Distinguished Race-Specificity of RpsSDB and RpsDW

**Figure 4:**
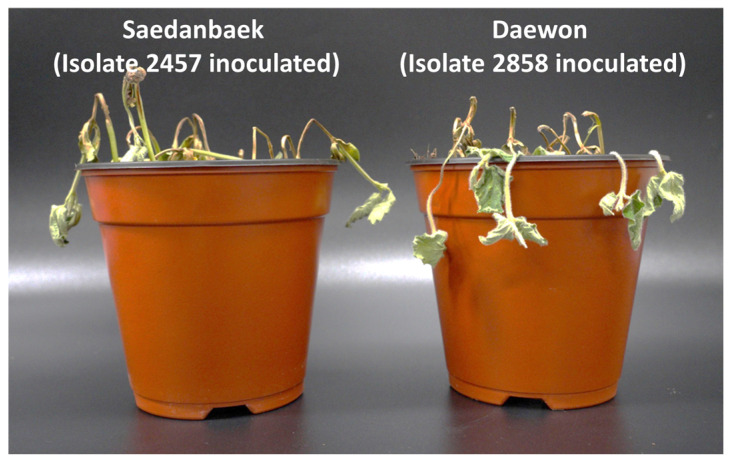
Susceptible reactions of Saedanbaek (**left**) and Daewon (**right**) following inoculation with isolates 2457 and 2858 of *Phytophthora sojae,* respectively.

Saedanbaek and Daewon were inoculated with isolates 2858 and 2457, and their race specificity was confirmed. Saedanbaek was resistant to isolate 2858 but susceptible to isolate 2457 (Figure 4). Daewon showed susceptibility and resistance to isolates 2858 and 2457, respectively (Figure 4). To confirm the reciprocal race-specific reactions of *RpsSDB* and *RpsDW*, 25 RILs harboring the Saedanbaek-resistant allele on chromosome 3 and 35 RILs that included the Daewon-resistant allele on chromosome 3 were inoculated with isolates 2858 and 2457, respectively. Daepung was susceptible to these two isolates. Saedanbaek and 25 RILs that were resistant to isolate 2858 were susceptible to isolate 2457 (Table 5). Similarly, Daewon and 35 RILs that were resistant to isolate 2457 were confirmed to be susceptible to isolate 2858 (Table 5). As all the tested RILs and the two resistant parents exhibited susceptibility to the tested isolates with 100% seedling death, race-specific resistance of the respective *RpsSDB* and *RpsDW* was confirmed, and either one could effectively protect against only one isolate.

## 3. Discussion

Characterization of valuable genetic sources for a target trait is the most important initial step in plant breeding. Previously, few Korean varieties have exhibited resistance to some selective *P. sojae* isolates, and two varieties, Daewon and Socheong2, have been genetically characterized for their inheritance of resistance to *P. sojae* isolates 2457 and 40412, respectively [24,48,49]. In the present study, the Korean high-protein variety, Saedanbaek, was used to analyze its *Rps*-mediated resistance to *P. sojae* isolate 2858 using the Daepung × Saedanbaek RIL population. Soybean resistance to isolate 2858 was investigated for the first time in this study. The majority of RILs were clearly segregated into either resistant (R) or sensitive (S) phenotypes following hypocotyl inoculation, and a single resistance gene locus (*RpsSDB*) was mapped to the 3.3–4.3 Mbp region of chromosome 3 via both single-marker ANOVA and linkage analysis (Figure 2, Table 4). This locus explained approximately 82% and 93% of the phenotypic variation with ANOVA and composite interval mapping, respectively, indicating that the resistance was controlled by a single major gene in a qualitative manner (Figure 2, Table 4). These high *R*^2^ or PVE values of the identified SNP strongly support that distinct phenotypes are clearly segregated in the mapping population and a strong association between phenotypic and genotypic data is quite evident. Also, the size of the mapping population (i.e., *N* = 72) is sufficient to identify a major locus controlling resistance to *P. sojae.*

The distribution of *Rps* genes appears to be uneven over 20 chromosomes; more than 20 *Rps* genes have been identified on chromosome 3 from different resistance sources [12,13,14]. This is comparable with another ‘hot spot’ of *Rps*, the approximately 53−56 Mbp region on chromosome 18, where *Rps4*, *5*, *6*, *12*, *JS*, and unnamed in cv. CheonAl partially overlapped or were physically separated based on their approximate positions [12,36,38,39,40,49,50]. Some of the *Rps* alleles in these clusters were roughly mapped by genetic analyses with small numbers of molecular markers and/or small sizes of mapping populations without further fine-scale mapping. Thus, it is unclear whether they all are indeed allelic to each other or belong to adjacent different loci. In the present study, Saedanbaek and Daewon exhibited reciprocal race-specific reactions with isolates 2858 and 2457. However, the two presumably-adjacent *Rps* genes were not fully defined for their physical positions due to lack of a few RILs with recombination breakpoints within the target interval, which facilitate a narrowing-down of the target interval as used in the fine mapping of *RpsWY* and *RpsGZ* [15,28]. Using the 180 K SNP data, haplotypes of this overlap were compared among the three parental varieties, though any feature determining the allelic relationship was not discovered. Given this results, genetic allelism tests should be performed using the F_2:3_ Daewon × Saedanbaek population and the two *P. sojae* isolates to confirm the allelic relationship between *RpsSDB* and *RpsDW*. Deep-depth sequencing analysis should assist to elucidate the relationship of the two *Rps* genes for further study. More accurate determination of these genes/alleles will aid in the marker-assisted selection for multiple *Rps*-stacked lines.

The NBS-LRR genes are the extremely large family of plant disease resistance genes and their clustered/tandemly-repeated nature or the evolutionary complexity has been well documented [32,33]. According to the gene annotation (Glym.Wm82.a2.v1), multiple copies of NBS-LRR- and STK-coding genes were commonly distributed in the two hotspot regions mentioned above. The intervals in the respective *RpsDW* and unnamed *Rps* of CheonAl included 14 and 9 genes [24,50]. Another *R*-gene *RpsUN1* was fine-mapped within a 151 kbp interval that harbored five candidate genes, including three predicted *R*-gene homologs [18,51]. *RpsHN* was also mapped to a 278 kbp region, where eight genes were annotated, including one STK gene [19]. The overlapping 403 kbp region between *RpsSDB* and *RpsDW* includes six NBS-LRR genes (Figure 3). Of the six genes, Glyma.03G034900 has been previously highlighted as a candidate gene for *P. sojae* resistance in *RpsHN* and *RpsYD25* [19,52]. The expression of this gene increased 12 h after *P. sojae* inoculation in Meng8206 (resistant), whereas it did not significantly change in LinMeng6-46 (susceptible) [19]. Nearby, slightly outside the overlapping region, Glyma.03G034600 was also identified as a candidate gene for *RpsUN1* based on fine mapping and gene expression analysis [51]. These *Rps* clusters will be interesting resources for studying the dynamics of the *R*-genes and understanding them will provide insights regarding the mechanism of *P. sojae* resistance.

The most significant marker, AX-90404495, was mapped approximately 25 and 20 cM from two flanking markers. As noted above, this is not a single SNP, but is representative of 28 co-segregating SNPs located over 3.3–4.3 Mbp. Considering the genomic distances to the flanking SNPs based on the reference genome (Glyma.Wm82.a2), the distances between the markers were notably large, implying that the recombination frequency might be high around this *R*-gene locus (Figure S1). Interestingly, high recombination frequencies were observed in the similar genomic region of chromosome 3 from Shuurei × Tosan-231 population [30]. The possible explanations for this result are as follows: (i) the small size of the mapping population may lead to a relatively biased recombination frequency in this region [53], (ii) chromosomal rearrangement or structurally variable regions by genotype may exist between the representative U.S. variety (i.e., Williams82) and non-U.S. variety [54], or (iii) unequal crossing over, a driving force of *R*-gene evolution, may increase the variability of this region [55]. These factors can result in an overestimated recombinant frequency, leading to prolonged marker intervals.

Genomic rearrangements or recombination, such as gene duplication, unequal exchange, abnormal chromosomal recombination, and gene conversion, have been reported as important mechanisms that affect the copy number of disease-resistant genes [55]. In soybeans, *Rps11,* an NBS-LRR gene that was subjected to map-based cloning, was derived from rounds of unequal crossover events, resulting in promoter fusion and LRR expansion [56]. Consequently, this change led to broad-spectrum resistance to 127 *P. sojae* isolates widely distributed across the USA. Copy number variations in the *Rhg1-b* allele of soybean have also been associated with resistance to the soybean cyst nematode (SCN) [57]. The susceptible varieties for SCN have one copy of the 31 kbp segment, which encodes resistant proteins for SCN, whereas the *rhg1-b* haplotype harbors 10 tandem copies with increased resistance to SCN. The maize *Rp1* locus is characterized as a complex region related to resistance to the fungus *Puccinia sorghi,* and some *Rp1* alleles are meiotically unstable, showing unequal exchange in this region [58]. Unequal exchange in the *Rp1* locus leads to variations in the repeat number of *Rp1*, resulting in altered disease resistance. These reports suggest that recombination events in repeated LRR genes may affect the number or structure of LRR genes, leading to changes in resistance and race-specific reactions.

The development of broad-spectrum disease-resistant varieties is an ideal goal of soybean breeding programs. Some loci have been associated with broad-spectrum resistance to several *P. sojae* isolates. *RpsYD25* identified in the Chinese soybean cultivar Yudou25 exhibited broad-spectrum resistance to *P. sojae* isolates with different pathotypes in China [59]. Among the 26 *P. sojae* pathotypes, *RpsYD25* showed resistance to 21 isolates with differing pathotypes. Another *Rps* locus, *Rps11*, in the soybean landrace PI 594527, showed broad-spectrum resistance to 16 *P. sojae* isolates [56]. Tightly linked or pyramided multiple *Rps* genes also facilitate to protect soybeans against a broad-range of *P. sojae* isolates [30,60]. As most of the tested Korean soybean varieties were susceptible to four *P. sojae* isolates, and some were resistant to only a few isolates [48], the pyramiding of multiple *Rps* alleles from different resistance sources is a desirable approach in developing new resistant varieties. For this, the identification of germplasms with resistance to diverse pathotypes should be prioritized.

In summary, a locus, named *RpsSDB*, resistant to *P. sojae* isolate 2858, was mapped in the 921 kbp region, which is a well-known *Rps* gene cluster harboring enriched NBS-LRR annotated genes, on chromosome 3 in the Daepung × Saedanbaek population. The physical position of *RpsSDB* partially overlapped with the previously detected *RpsDW* in the 402 kbp interval, which harbors six annotated NBS-LRR genes. Interestingly, *RpsSDB* and *RpsDW* reciprocally showed race-specific resistance to either isolate 2457 or 2858, implying that they may be two allelic forms of one gene or two different genes. The current genetic mapping and haplotype comparison did not differentiate the chromosomal positions of the two *Rps* from those different varieties. In future studies, high-throughput sequencing, relative expression analysis, or genetic tests for allelism using a cross of Saedanbaek × Daewon will be able to clarify whether *RpsSDB* and *RpsDW* are two different genes adjacently located in the genome. The results of this study will assist soybean breeders in developing soybean lines with broad spectrum resistance to *P. sojae* infection.

## 4. Materials and Methods

### 4.1. Plant Materials

Daepung, Saedanbaek, and 72 RILs derived from crosses between Daepung and Saedanbaek were used in this study. Daepung is an elite Korean cultivar with lodging tolerance [61], and Saedanbaek is widely used in making tofu because of its high protein content and resistance to bacterial pustules [62]. Both parental lines exhibited white flowers, yellow seeds, and determinate growth habits. In our preliminary experiments, two soybean varieties, Daepung and Saedanbaek, were classified as susceptible or resistant to *P. sojae* isolate 2858 (Figure 1). In 2019, the RIL population was obtained from Dankook University in Cheonan, Republic of Korea. To propagate the seeds, the F_6_-derived RIL population was grown in a research field at the Chungnam National University, Daejeon, Republic of Korea. In this study, two parents and 72 RILs were used in subsequent experiments.

### 4.2. Evaluation of Resistance to P. sojae Isolate 2858

The *P. sojae* isolate 2858 was originally collected in Gyeonggi-do and provided by the Department of Southern Area Crop Science, National Institute of Crop Science, Rural Development Administration, Miryang, Gyeongsangbuk-do, Republic of Korea. A single oospore of the isolate 2858 was re-isolated from the original isolate for uniformity of results and then it was stocked for the following phenotypic assays. The inoculum was prepared as previously described [63]. Briefly, *P. sojae* was incubated in V8 juice medium for 7 days. The V8 medium, which was fully covered by *P. sojae* mycelia, was macerated using a 50 mL syringe and transferred into a 10 mL syringe before inoculation. Ten to twelve seedlings of each genotype were grown in plastic pots and inoculated using the hypocotyl inoculation technique when they were 7 days old after sowing [63]. A short slit (about 1 cm) was made using a scalpel on the upper part of the hypocotyl, and 0.2–0.4 mL of a mycelial slurry was placed inside the slit. The inoculated plants were maintained under dark and humid conditions (>90% relative humidity, 25 °C) for 16 h, and moved into a plant growth chamber (14/10 h of light/dark condition at 25 °C with >70% relative humidity). Seven days after inoculation, the percentage of dead seedlings was calculated and (2)the reaction of each RIL was determined in a categorical manner based on the percentage of dead seedlings; resistant (R) for ≤20%, intermediate (I) for 20–80%, or susceptible (S) for ≥80%. The phenotypic assay was repeated thrice.

### 4.3. DNA Extraction and Single Nucleotide Polymorphism (SNP) Genotyping

The genomic DNA of parents and RILs was extracted using cetyltrimethylammonium bromide (CTAB) [64] and genotyping was performed using Axiom^®^ 180K SoyaSNP arrays (Affymetrix, Santa Clara, CA, USA) [65]. The SNP data were preprocessed as follows: in total, 169,028 high-quality SNPs were obtained from the SNP array and 142,036 undefined or non-polymorphic SNPs were removed. Additionally, 862 SNPs were eliminated because of ≥10% missing. In total, 26,130 SNPs were included in the final dataset for subsequent genetic analyses. 

### 4.4. Goodness-of-Fit Test and Single-Marker Analysis of Variance

The segregation ratio for *P. sojae*-resistant reactions was investigated in the RIL population and the Chi-square (χ^2^) test was used. Marker-trait associations were analyzed using single-marker ANOVA using the lme4 package 1.1.31 [66] in R-4.3.2 [67]. Bonferroni correction was used to determine a conservative threshold for *p*-value (i.e., *p* < 3.8 × 10^−7^) using the following equation: α/N, where α is 0.01 and N is the number of SNPs.

### 4.5. Construction of Genetic Map and Linkage Analysis

A genetic map of the Daepung × Saedanbaek RIL population was constructed using IciMapping version 4.2 following the users’ instructions [68]. Of the 26,130 SNPs, redundant SNPs were filtered using the BIN function. Filtered SNPs were grouped and ordered using the MAP function. Linkage analysis was conducted using the BIP function, where the average values of the percentage of dead seedlings over replicates were used as the phenotype. With mapping steps of 1 cM, a 1000-permutation test was performed to determine the LOD threshold at *p* < 0.05 [69]. Graphical presentations of the genetic map and QTL plots of LOD scores were produced using MapChart 2.32 [70].

### 4.6. Cross-Validation of the Allelic Relationship between Saedanbaek and Daewon

As described in Section 4.2, additional *P. sojae* inoculation assays were performed using the hypocotyl inoculation technique [63] to determine whether two different genes were located adjacently and conferred resistance to the respective isolate in each resistant variety. Saedanbaek, and 25 RILs derived from a cross between Daepung and Saedanbaek, which showed resistance to isolate 2858, were inoculated with isolate 2457. Similarly, Daewon and 35 RILs derived from a cross between the Daepung and Daewon populations that exhibited resistance to isolate 2457 were also tested using isolate 2858.

## Figures and Tables

**Table 1:** Goodness-of-fit test for segregation ratios in the 72 recombinant inbred lines (RILs) of the Daepung × Saedanbaek population following inoculation with *Phytophthora sojae* isolate 2858.

Parents and RILs	Observed ^1^			Expected ^1^			Goodness-of-Fit	
	R	I	S	R	I	S	χ^2^	*p*
Daepung (P1)			50					
Saedanbaek (P2)	50							
Daepung × Saedanbaek (RILs)	25	4	43	34.9	2.25	34.9	6.05	0.05

^1^ R, resistant reaction; S, susceptible reaction; I, intermediate reaction.

**Table 2:** Twenty-eight single nucleotide polymorphisms (SNPs) significantly associated with resistance to *Phytophthora sojae* isolate 2858.

Chr ^1^	Position (bp) ^2^	SNP ID	Daepung Allele (S)		Saedanbaek Allele (R)		Adjusted	*R* ^2 4^
			Genotype	Frequency	Genotype	Frequency	*p*-Value ^3^	
3	3,373,644	AX-90419199	AA	0.64	GG	0.33	1.54 × 10^−22^	0.81
3	3,395,315	AX-90404495	TT	0.64	CC	0.36	4.41 × 10^−23^	0.82
3	3,403,744	AX-90448600	CC	0.64	TT	0.36	4.41 × 10^−23^	0.82
3	3,403,812	AX-90372396	AA	0.64	GG	0.36	4.41 × 10^−23^	0.82
3	3,417,978	AX-90354028	CC	0.64	TT	0.36	4.41 × 10^−23^	0.82
3	3,423,161	AX-90380038	TT	0.64	CC	0.36	4.41 × 10^−23^	0.82
3	3,458,537	AX-90524133	CC	0.64	TT	0.36	4.41 × 10^−23^	0.82
3	3,464,766	AX-90414569	TT	0.64	GG	0.36	4.41 × 10^−23^	0.82
3	3,464,955	AX-90387750	AA	0.64	GG	0.36	4.41 × 10^−23^	0.82
3	3,484,755	AX-90378648	TT	0.63	GG	0.36	4.41 × 10^−23^	0.82
3	3,485,682	AX-90470694	TT	0.64	CC	0.33	9.62 × 10^−19^	0.76
3	3,517,886	AX-90449575	AA	0.64	CC	0.36	4.41 × 10^−23^	0.82
3	3,554,469	AX-90388258	TT	0.63	AA	0.36	3.68 × 10^−16^	0.71
3	3,695,759	AX-90317052	GG	0.63	AA	0.37	4.41 × 10^−23^	0.82
3	3,709,588	AX-90394026	CC	0.63	TT	0.36	4.41 × 10^−23^	0.82
3	3,759,276	AX-90440228	CC	0.64	TT	0.36	4.41 × 10^−23^	0.82
3	3,847,841	AX-90375748	AA	0.63	GG	0.37	4.41 × 10^−23^	0.82
3	3,849,443	AX-90499181	GG	0.64	AA	0.36	4.41 × 10^−23^	0.82
3	3,855,656	AX-90467840	CC	0.63	TT	0.37	4.41 × 10^−23^	0.82
3	3,875,061	AX-90449384	TT	0.64	CC	0.34	2.18 × 10^−22^	0.81
3	4,272,521	AX-90417885	GG	0.63	AA	0.37	4.41 × 10^−23^	0.82
3	4,272,894	AX-90347629	TT	0.63	CC	0.37	4.41 × 10^−23^	0.82
3	4,277,380	AX-90310078	TT	0.63	CC	0.37	4.41 × 10^−23^	0.82
3	4,283,885	AX-90465452	TT	0.64	AA	0.36	4.41 × 10^−23^	0.82
3	4,284,091	AX-90328472	CC	0.64	TT	0.30	3.98 × 10^−20^	0.78
3	4,291,232	AX-90331552	TT	0.63	CC	0.37	4.41 × 10^−23^	0.82
3	4,291,566	AX-90307867	AA	0.63	TT	0.37	4.41 × 10^−23^	0.82
3	4,295,128	AX-90317436	AA	0.63	GG	0.37	4.41 × 10^−23^	0.82

^1^ Chromosome. ^2^ Physical positions are based on soybean genome Glyma2 (http://soybase.org). ^3^ Adjusted *p*-value was obtained using Bonferroni correction (*p* < 1.91 × 10^−6^). ^4^ Phenotypic variance explained by each marker.

**Table 3:** Information regarding the genetic map of the Daepung × Saedanbaek population.

Chr. ^1^	Total Length (cM) (a)	No. of Total SNP Markers	No. of Unique Loci (b) ^2^	Avg. Marker Interval (cM) (a/b) ^3^
1	87.4	1485	58	1.5
2	100.4	1335	63	1.6
3	127.0	1165	48	2.6
4	62.9	1338	46	1.4
5	68.6	1358	51	1.3
6	79.2	1583	61	1.3
7	70.2	1094	46	1.5
8	99.2	1350	52	1.9
9	54.2	1198	33	1.6
10	74.4	1515	52	1.4
11	73.9	498	40	1.8
12	49.4	987	36	1.4
13	92.2	1704	43	2.1
14	66.7	1092	47	1.4
15	95.4	1936	57	1.7
16	59.7	1281	39	1.5
17	92.3	709	41	2.3
18	99.6	1822	59	1.7
19	66.2	1594	44	1.5
20	67.6	1086	47	1.4
Total	1586	26,130	963	

^1^ Chromosome. ^2^ Actual number of loci where markers are positioned in the genetic map. ^3^ Average interval between loci, which was calculated by dividing total length (cM) by no. of unique loci.

**Table 4:** A genomic region identified for resistance to *Phytophthora sojae* isolate 2858 in the Daepung × Saedanbaek population.

Chr. ^1^	Position (bp) ^2^	Flanking Markers	LOD ^3^	PVE (%) ^4^
3	3,373,644…4,295,128	AX-90419199…AX-90317436	38.7	93.0

^1^ Chromosome. ^2^ Physical position in bp based on the genome version Glyma2. ^3^ Logarithm of odds (LOD). ^4^ Phenotypic variance (%) explained by the QTL.

**Table 5:** Response of Saedanbaek, Daewon, and RILs of two populations following inoculation with *Phytophthora sojae* isolates 2858 and 2457.

Parents and RILs	Reaction to *P. sojae* Isolates Following Inoculation	
	2858	2457
Daepung	S	S
Saedanbaek	R	S
RILs ^1^ (*N* = 25)	All R	All S
Daepung	S	S
Daewon	S	R
RILs ^2^ (*N* = 35)	All S	All R

^1^ The RILs with resistance to the isolate 2858 from the Daepung × Saedanbaek population. ^2^ The RILs with resistance to the isolate 2457 from the Daepung × Daewon population.

## Data Availability

Data are contained within the article and Supplementary Materials.

## Supplementary Materials

The following supporting information can be downloaded at: https://www.mdpi.com/article/10.3390/plants12233957/s1, Figure S1: Collinearity between genetic (cM) and physical (Mbp) positions of the mapped SNPs; Table S1. List of candidate genes in the *RpsSDB* and *RpsDW* region.
