# Supplementary figures and images for: Soybean Variety Saedanbaek Confers a New Resistance Allele to Phytophthora sojae
Source: Plants (Basel). 2023 Nov 24;12(23):3957. doi: 10.3390/plants12233957 (PMC10707759; doi:10.3390/plants12233957)

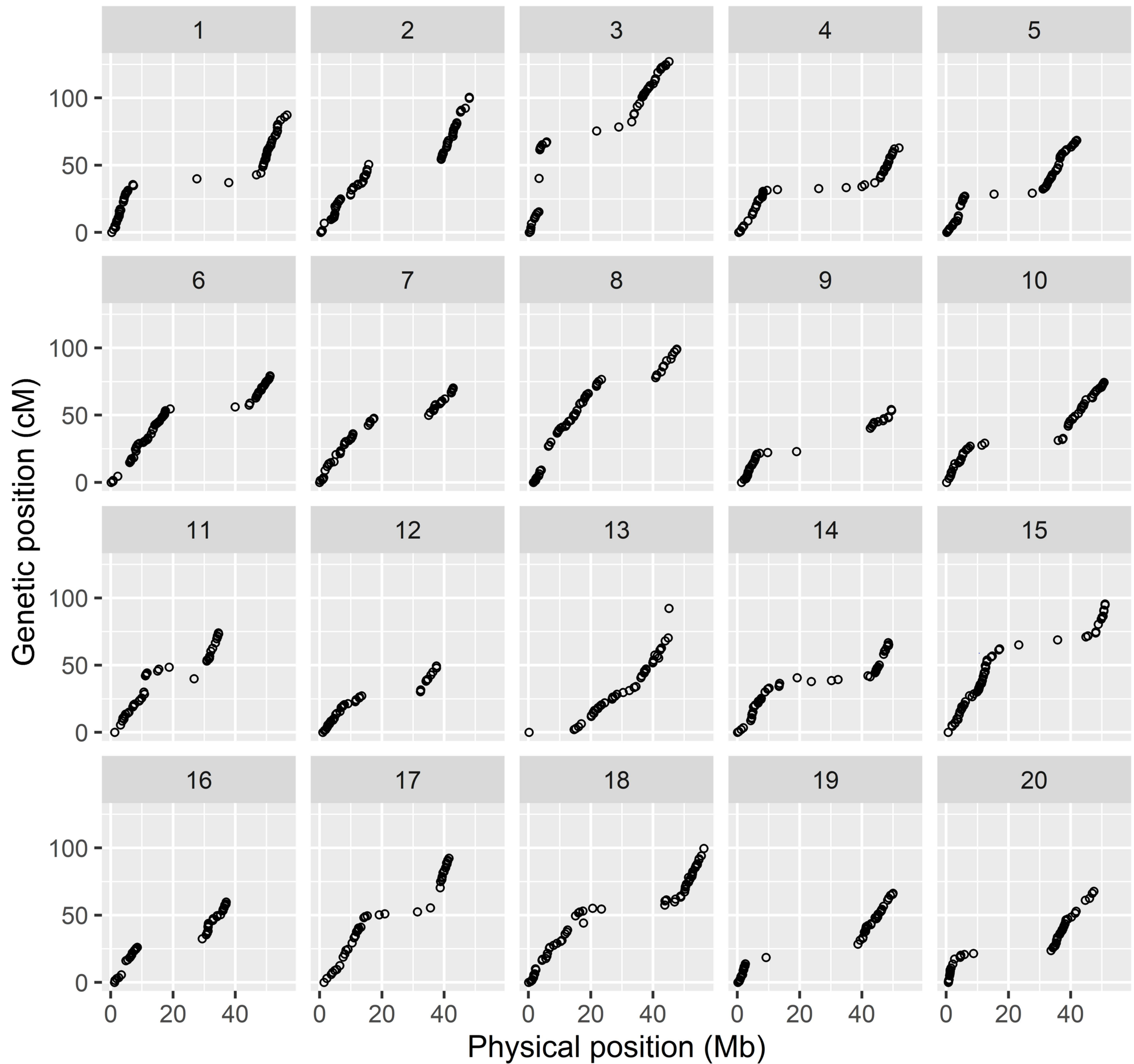

**Figure S1.** Collinearity between genetic (cM) and physical (Mbp) positions of the mapped SNPs.

Supplement: Supplementary file 1 [file plants-12-03957-s001.zip › Figure S1.pdf]
